# Adaptive adjustment to the needs of families caring for children and adolescents with physical disabilities in north-eastern Tanzania: a grounded-theory study

**DOI:** 10.1080/16549716.2024.2354009

**Published:** 2024-06-04

**Authors:** Elia Asanterabi Swai, Haleluya Imanueli Moshi, Sia Emmanueli Msuya, Marie Lindkvist, Ann Sörlin, Klas Göran Sahlen

**Affiliations:** a Department of Physiotherapy, Kilimanjaro Christian Medical University College (KCMUCo), Moshi, Tanzania; b Department of Epidemiology and Global Health, Umeå University, Umeå, Sweden; c Department of Community Medicine and Rehabilitation, Umeå University, Umeå, Sweden; d Community Health Department, Institute of Public Health, Kilimanjaro Christian Medical University College, Moshi, Tanzania; e Department of Epidemiology and Biostatistics, Institute of Public Health, Kilimanjaro Christian Medical University College, Moshi, Tanzania; f Department of Community Medicine, Kilimanjaro Christian Medical Centre, Moshi, Tanzania

**Keywords:** Paediatrics, public health, physical disabilities, challenging needs, adaptive adjustment

## Abstract

**Background:**

Family interactions, which are always multi-faceted, are complicated further by family members with disabilities. In resource-poor settings, policies and programmes that address the needs of and challenges faced by families are often inaccessible or unavailable. Approximately 13% of the families in Tanzania have at least one member with a disability, yet family-centred research on caring for disabled children and adolescents is scarce in this context.

**Objective:**

The aim is to explore the needs and challenges faced by families that care for children and adolescents with physical disabilities in the Kilimanjaro Region of north-eastern Tanzania.

**Methods:**

This qualitative study had a constructivist grounded-theory design. In-depth interviews, using a semi-structured interview guide based on the social-capital framework, were conducted with 12 female participants aged between 24 and 80. A conceptual model of family needs, inspired by Maslow’s hierarchy of needs, informed the analysis.

**Results:**

Challenging needs were grouped into five categories, which were linked to Maslow’s hierarchy of needs and related to the central concept of ‘adaptive adjustment’: (1) ‘barely surviving’; (2) ‘safety needs in jeopardy’; (3) ‘sociocultural protection’; (4) ‘self-esteem far beyond reach’, and (5) ‘dreaming of self-actualisation’.

**Conclusion:**

Families caring for children and adolescents with physical disabilities in north-eastern Tanzania have needs that extend beyond the available and accessible resources. Families can adjust and adapt by avoiding certain situations, accepting the reality of their circumstances and exploring alternative ways of coping. A sustainable support system, including social networks, is essential for meeting basic needs and ensuring safety.

## PAPER CONTEXT

- **Main findings:** The challenges associated with caring for children and adolescents with disabilities are related to various environmental and social barriers, any of which can significantly impact the wellbeing of all family members.
- **Added knowledge:** In the context of a study conducted in north-eastern Tanzania, both nuclear and extended family networks were found to be valuable sources of support for these families.
- **Global health impact for policy and action:** Families must take considerable steps to strengthen their sociocultural support systems while, the support of the government and other stakeholders is crucial in addressing the challenges faced by families caring for children and adolescents with disabilities.

## Background

Globally, one billion individuals (16% of the world population) have some form of disability, and around one-third of these are children and adolescents [1,2]. In this study, ‘disability’ refers to physical deficits in bodily structure and function, as well as difficulties in performing activities and participating in life situations [3,4]. Many children and adolescents with physical disabilities live in resource-poor settings where essential healthcare, such as rehabilitation and social services, is limited [1,5]. According to UNICEF’s ‘Situation analysis of children and young people with disabilities in mainland Tanzania and Zanzibar’, the prevalence of disability among children and young people (aged 5 to 24 years) was 2.3% in 2021 [6,7]. In Tanzania, 13.2% of the families have at least one member with a disability.

The family unit is a sophisticated, interdependent, and interactive system, wherein the needs and experiences of all members are shared [8]. Children and adolescents with physical disabilities have special needs that require specialised resources [9–11]. Raising a child with a disability imposes a financial burden on a family, and creates multiple challenges related to the complexities of care [10,12]. Time for carers, typically the mother, to support the household financially is often limited [12–15]. In the absence of sustainable support, some families resort to selling assets to meet needs, and in extreme cases, succumb to severe poverty [15,16]. Financial and social support for resource-poor families is crucial [17–19]. In many low- and middle-income countries, including Tanzania, laws designed to address some of these barriers are often not implemented effectively [19–21].

In Tanzania, a policy supporting the families of people with disabilities has been in place since 1981. The policy explicitly stipulates that families, relatives, and the government are responsible for providing care to family members with disabilities [22]. The Disability Acts of 2004 and 2010 specifically acknowledged the need to support service provision for families who care for children with disabilities [22,23]. Nonetheless, families in Tanzania struggle to meet the needs of their physically disabled children. This leads to hardship, especially for carers [24]. Tanzania’s population has nearly doubled since 2004, when the Act dedicated to people with disabilities was passed [25]. There is a need for policy implementation with a focus on disabled people and their families.

Knowledge concerning the needs of families caring for children and adolescents with physical disabilities in low-income countries is limited. Few qualitative studies have been conducted on childhood disability from the family perspective [11]. Improving knowledge and understanding in this space will benefit all stakeholders. The aim of this study was to explore the needs and challenges faced by families caring for children and adolescents with physical disabilities in the Kilimanjaro Region in north-eastern Tanzania.

## Methods

### Study design

In this qualitative paper we present a constructivist grounded-theory design [26]. Grounded theory aims to develop a theory ‘grounded’ on empirical data [26–28]. This method is appropriate when the theoretical underpinnings of the investigation are underdeveloped [29]. The grounded-theory approach facilitates the understanding of a particular phenomenon (in this case family needs) within an interactive process (adjustment) [30]. Constructivist grounded theory acknowledges that reality is socially constructed and that multiple interpretations can coexist [26,31].

### Study setting

The study was conducted in the Kilimanjaro Region in north-eastern Tanzania, which has a population of approximately 1.9 million [32]. The region possesses a unique cultural blend of two main resident tribes: the Chagga (comprising nearly 10 dialects) and the Pare. Administratively there are seven districts: Moshi Municipal, Moshi Rural, Rombo, Hai, Siha, Same, and Mwanga. The Pare tribe is found in Same and Mwanga, and the Chagga in the remaining districts. The primary economic activities are small-scale agriculture and tourism.

The majority of the Kilimanjaro Region’s population resides in typical rural African areas characterised by poor economic conditions, informal transportation systems, and inadequate road infrastructure. Health and social services, particularly those related to rehabilitation, are scarce [33]. Primary healthcare facilities in rural areas have few or no rehabilitation services. The available rehabilitation services are mainly located in a few specialised healthcare facilities in urban areas, which are inaccessible to and unaffordable for poor rural families.

### Recruitment

A previous survey on disability-related needs informed the selection of the participants [33]. The survey showed that more than two-thirds (77%) of children are not covered by health insurance, more than one-third (40%) of children and adolescents with disabilities had lost access to rehabilitation services, and almost a quarter (24%) had never had access to any such service. The recruitment process considered variations in access to health insurance and rehabilitation. We recruited participants with diverse characteristics in order to gain a deeper understanding of their needs and challenges (Table 1). In the initial stage, EAS contacted the participants to explain the purpose of the study and schedule the interviews. During the interview visits, EAS provided the participants with informed consent forms and a detailed description of the study. The study was ethically approved by the local institutional review board, and all participants verified their voluntary participation by signing the consent form (see ‘Ethics and Consent’ section at the end of the text).

**Table 1.** Descriptive summary of the participants.

Participant’s age (years)	Marital status	District of residence	Residence area category	Child age (years)	Child sex	Relationship	Health-insurance coverage	Rehabilitation access
48	Married	Moshi Municipal	Urban	9	Male	Biological mother	Yes	Lost access to rehabilitation services
36	Married	Hai	Urban	7	Female	Biological mother	Yes	Lost access to rehabilitation services
54	Married	Moshi Municipal	Urban	18	Male	Biological mother	Yes	Yes
80	Widowed	Moshi Rural	Rural	6	Female	Grandmother	No	Lost access to rehabilitation services
33	Single	Moshi Rural	Rural	4	Female	Biological mother	No	Yes
43	Single	Same	Rural	6	Male	Biological mother	No	Yes
45	Separated	Same	Urban	9	Female	Biological mother	No	Yes
30	Divorced	Hai	Rural	9	Male	Biological mother	No	Lost access to rehabilitation services
52	Separated	Moshi Rural	Rural	18	Female	Biological mother	No	Never
38	Married	Rombo	Rural	9	Male	Biological mother	No	Lost access to rehabilitation services
49	Single	Moshi Rural	Rural	12	Female	Biological mother	No	Never
39	Married	Rombo	Rural	10	Male	Biological mother	No	Never

### Participants

The participants (carers for children and adolescents with physical disabilities) were purposively sampled. All were female primary carers aged between 24 and 80 years. In African culture, particularly in rural areas, the husband heads the household, and the mother looks after the children. In an effort to be respectful and culturally sensitive, the researchers considered inviting fathers to participate in the interviews. However, after being informed of the aims and purpose of the study, fathers willingly trusted mothers to participate alone in all interviews. Single mothers (separated, divorced, or never married) and one grandmother were also invited. Saturation was considered to be the point at which the inclusion of new participants ceased to yield novel insights; it was assumed after the tenth interview that this had been reached, but this assumption was verified by conducting an additional two interviews with participants who varied in terms of age, area of residence, and child age, sex, diagnosis, etc [26,34].

### Data collection

Semi-structured in-depth interviews were undertaken from February to June 2022 by EAS in Swahili in the participants’ homes. The interview guide was revised based on feedback from a pilot interview. This was transcribed and reviewed by EAS, who is fluent in both Swahili and English, and KS. The interviews established rapport through a series of general questions, which was followed by more specific content themes [35]. The social-capital framework of Pierre Bourdieu informed the design of the data-collection tool [36]. The interviews lasted between 59 and 135 min, and were digitally recorded, transcribed verbatim, and translated from Swahili to English by EAS. To ensure the accuracy of the translations, the co-author HIM (also fluent in Swahili) cross-checked the translations. The field observation notes were recorded in a logbook by EAS. Data collection and the initial open coding were conducted simultaneously.

The first author is a physiotherapist with a background in working with community programmes for children with disabilities in Tanzania. This contextual experience was important for both the interactions with the participants and enhancing theoretical understanding during the data-collection process. Together, the co-authors brought diverse cultural and professional backgrounds in public health, adolescent health, physiotherapy/rehabilitation, and qualitative research methodologies.

### Data analysis

**Figure 1.**
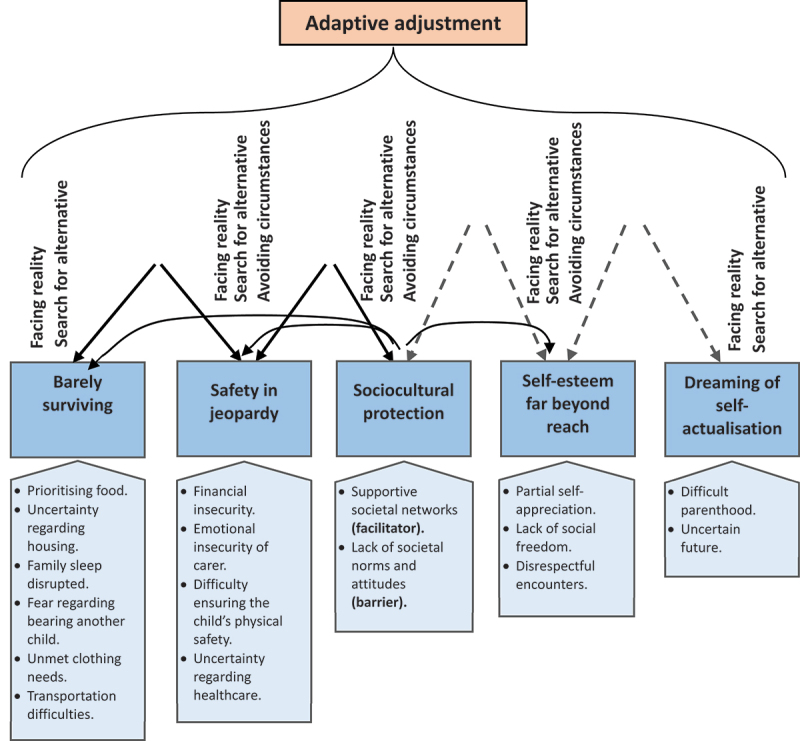
The theoretical model, delineating the core category of ‘adaptive adjustment’. This comprised several categories and subcategories.

The data analysis involved open, selective, axial, and theoretical coding using Maslow’s hierarchy of needs as the sensitising concept (Table 2). The analysis process involved continuous comparisons, aided by memos, field notes, and transcripts. Open Code software Version 4.03 [37] was used to facilitate coding and transcript organisation. The open codes were exported to an Excel spreadsheet for further synthesis. The first three interviews were coded line by line, assigning codes to each sentence, phrase, or in-vivo code by EAS and KS. During selective coding, the codes relevant to the research question were selected and referred to the text. Codes with similar patterns were clustered to form categories. Axial coding examined the properties of the categories, which were unpacked into subcategories. The sensitising concept aided in theoretical coding and resulted in five categories highlighting human needs and challenges that were examined for relationships. The key questions asked were what was taking place and why, how, and with what consequences this happened. The core category of ‘adaptive adjustment’ emerged and appeared to explain how families navigate challenging needs, as illustrated in Figure 1. Maslow’s hierarchy of needs theory informed the narrative constructed within this research because of its ability to explain the fulfilment of human needs. The refinement of the modal used a consistent comparison approach by comparing codes with codes, codes with emerging categories, categories with categories, and emerging theory with the literature [38,39]. The final modal in Figure 1 shows the dynamics of families in relation to addressing the needs of families caring for children and adolescents with physical disabilities.

**Table 2.** Examples of the coding process.

Text	Line-by-line coding	Selective coding	Axial coding	Theoretical coding
… taking care of this child is a huge cost, that is, it is very high. I tell you very clearly to this day he drinks fresh milk …	Caring for a disabled child is costly. Caring comes with costs. The child requires special nutritional attention. Food is expensive. Additional nutritional needs. An older child needs milk, like a small baby.	Nutritious food needed + Higher expenses for food + Additional food needed	Family prioritising food needs	Barely surviving
… with the suffering I went through with this child, I just don’t want to have a baby again. I thank God that he helps and protects me so that I can raise her …	The decision to have more children is affected. Suffering related to caring for a disabled child. Not wishing to have another child. Hesitant to have another child. Not ready to have more children. Seeking support from God. All focus on raising the disabled child.	Hesitant to bear more children + Afraid to suffer with another child + Worried about having children	Fear of bearing more children	

### Theoretical framework

In 1943, Maslow presented his five-tier classification of human needs in the form of a hierarchy [40], which has since been applied in a wide variety of contexts. The three lowest levels are the ‘deficiency needs’, consisting of basic, safety, and love/belonging; the upper two are the ‘growth needs’ of self-esteem and self-actualisation. Maslow stated that the lower needs must be completely satisfied before the upper needs can be addressed [41].

## Results

### Overview

The analysis resulted in five categories: ‘barely surviving’, ‘safety needs in jeopardy’, ‘sociocultural protection’, ‘self-esteem far beyond reach’, and ‘dreaming of self-actualisation’. These categories relate to the basic tangible and intangible aspects of the families’ needs. The subcategories relate to specific issues regarding unmet needs, challenges, and hardships. The core category of ‘adaptive adjustment’ explains how the families navigate the challenges associated with addressing their needs. Only one category (sociocultural protection) included both facilitator and barrier properties (Figure 1).

The darker blue boxes represent the needs of families who care for children and adolescents with physical disabilities, which were categorised into five major areas. The narrative constructed within this research for the categories and subcategories corresponds to Maslow’s hierarchy of needs. The lighter blue boxes represent subcategories, such as basic life necessities, resources, and desired position in society, and relate to how families fulfil their needs; using the constant comparison approach and Maslow’s hierarchy of needs theory, it was shown that these needs are not fulfilled in relation to the broader hierarchy. Each category was named to reflect the dynamics and challenges experienced in fulfilling each need. The needs are interrelated, as evidenced by the black arrows that connect the blue boxes. For example, unmet clothing needs, uncertainty regarding housing, and prioritising food all require financial resources, expressed as ‘financial insecurity’. The absence or insufficiency of resources is illustrated by the gaps between the arrows at their peaks, which relate to how challenging it is to address one level before fully addressing the next. Thus, families struggle to satisfy one category of needs (going up) and move onto another without assurance of stable fulfilment as a result of absent or insufficient resources, i.e. ‘barely surviving’. The dashed black arrows of self-esteem and self-actualisation symbolise disconnect and an unclear path to fulfilment. These dashed arrows also extend higher, suggesting that these needs are more challenging to fulfil. Belonging to a family or social network is crucial to families securing their basic needs, as indicated by the horizontal lines extending from the ‘sociocultural protection’ category. This encompasses support and resources from both the family and society, such as daily necessities, moral support, and relieving some of the care burden of the primary carer. Societal barriers (‘unsupportive encounters’) affect self-esteem and widen the gap in fulfilling needs. Families address their needs concurrently, struggling back and forth, and constantly adjust in order to adapt to situations. At each level, families adapt by acceptance (‘facing reality’) or hiding away/denying (‘avoidance’) and adjust by searching for alternatives.

### Barely surviving

‘Barely surviving’ refers to families constantly experiencing unfulfilled needs, and pertains to the subcategories of *prioritising food, uncertain housing possibilities, family sleep disrupted, fear of bearing another child, unmet clothing needs*, and *transportation barriers*. Although transportation is not part of Maslow’s hierarchy of needs theory, it is essential to fulfilling basic needs. Families caring for physically disabled children seek to meet their basic needs with minimal resources, and face inadequate access to essential services (healthcare, education, and transportation), insufficient resources, and high costs associated with food. The participants described the hardships faced in sourcing food for their families:

I think the diet is not enough because she is not healthy. If you go to the hospital, they tell the child to eat at least five times daily, but I find I cannot mix those foods or afford five meals. (Mother of a four-year-old girl)

The findings show that housing was felt to be a very problematic issue. The participants described difficulties paying rent, living in substandard houses in poor conditions and feeling uncertain about securing housing throughout the year. A single mother who relied on informal employment to support her nine-year-old daughter said:

There was a time when I could not pay rent here in town, so I kept my stuff as collateral and begged the landlord for an additional two months. The landlord was kind and gave me another chance.

Family members faced disrupted sleep because their disabled children woke them up several times at night. Urine-absorbing diapers were not affordable, and some had to change their child’s clothing during the night. Mothers were afraid of bearing more children because of limited family resources and the fear of having another child with a disability. The need for clothing was consistently unmet for most children and adolescents, and not seen as a priority.

The transportation barrier is operationalised here as a basic need. Transport is crucial because of the need to travel for services. Transportation difficulties increase as children grow and become heavier, exacerbated by limited and often crowded public transportation. Costs are also a barrier for families.

Transportation is a big challenge because it requires private transport, not public. On public transport, he will stretch himself, stiffen and hurt himself. […]One day he pushed himself hard and sustained a wound [on the public bus], which left this mark on his body. Look here [points at the scar]. (Mother of an 18-year-old boy)

### Safety needs in jeopardy

Viewed through the lens of Maslow’s hierarchy of needs, ‘safety’ encompasses *financial insecurity, emotional instability, difficulty ensuring the child’s physical safety*, *and uncertain healthcare*. The participants described the financial burden of meeting basic and disability-related needs as often being overwhelming for families. Furthermore, the emotional wellbeing of family members is constantly disturbed. The absence of husbands following divorce or separation, which often occurs due to a belief that a child’s disability is solely inherited from the mother, increases mothers’ emotional and physical vulnerability. The concerns expressed by the participants are also tied to the safety and wellbeing of their physically disabled children. Emotional insecurity is prominent in families with disabled adolescent girls, as described by the mother of an 18-year-old female:

I am very worried and don’t know what to do. The girl is more vulnerable and in danger than the boy. Because hasn’t she already become an adult? So now it means other things [referring to relationships, sex, and pregnancy] will happen if she finds a young man.

Healthcare presents many challenges, including limited access to services, poor-quality care, and high costs. Medications, exercise therapies, devices, and health insurance are all necessities, yet are often unavailable, inaccessible, or unaffordable. Unfortunately, the provision of healthcare services, including health insurance, is often discriminatory, as one mother of a nine-year-old girl heartbreakingly narrated:

I have gone to register her for health insurance twice, but children like her do not get insured. They said broken cars couldn’t be insured. Yes, I went to their office myself.

### Sociocultural protection

This category includes both facilitator and barrier properties. Being accepted by society is a vital part of navigating challenging needs. Culture also plays a significant role in the nuclear and extended family’s support network. Love, in the form of nuclear-family cohesion and extended family networks, provides a sense of safety for children and adolescents with physical disabilities. The presence of love and affection in the family facilitates the fulfilling of basic needs, as reported by the mother of a nine-year-old boy:

He is everything to us, his siblings, and all in the house. Our child comes first. So, he feels loved and cared for. I think that love and affection have given us the energy to care for him up to now.

In addition, positive encounters enhance acceptance and empower families to cope with their difficulties. The mother of a 10-year-old boy spoke of the way a positive encounter changed the situation in their neighbourhood.

The situation [discrimination] reached a point that scared us and made us unhappy. So, we used to hide the child inside the house because of fear. One of our priests advised us to first accept the child, tell ourselves that the child is a gift, and take the child outside so that people around can get used to him. So, we started bringing the child outside, and other children came to play with him in the evening. Now they are used to him, most of them like him and come to play with him regularly.

Having reliable nuclear and extended-family support enables families to address their needs and challenges positively. Adding any consistent/inconsistent support over this family asset facilitates empowerment and resilience. An example of blended support was described by the mother of an 18-year-old boy who has stable nuclear family support, is engaged in a small microfinance group, and is part of a network run by a faith-based NGO for disabled children:

The condition of the house had been bad since the birth of the [disabled] child […] one of my children said ‘mother, the house is very dirty, let’s paint it’. My eldest son has been very supportive, he has set aside a budget for his younger brother who has a disability, he has been very helpful.

Going to the KIKOBA [small social-microfinance group] is not in order to get rich. I’m engaged to manage the small things. Meeting with the group, even thoughts from peers, can change the loneliness you have at home, and you can change your mindset and laugh. We started it because we can help each other with the small things […] For example, I didn’t have running water at home. When I got into the KIKOBA I was able to borrow money and get water in my house.

Most of the participants showed the expectation of and hope for formal governmental support, even though it was absent for all families. The mother of a six-year-old boy said the following:

We ask the government to consider us families of children with disabilities, to give friendly small loans with small or no interest so that we can do something that can allow us to stay closer to the child. There are subsidies for families living in difficult conditions from TASAF [Tanzania Social Action Fund – a governmental support programme for poor families] that are often given to people who need it less, who for example can work and have fewer problems.

The social and cultural context facilitates the provision of needs through unstructured support from neighbours and community groups. The mother of an 18-year-old physically disabled girl living in a village said that belonging to a religious community supports her adolescent’s basic needs:

The church community gathering where we pray during the week certainly helps me. They provide support to families in need by providing soap and sugar regularly. They offer a particular package [money] that supports my child’s needs.

Believing in God is another contextual, cultural protection that is perceived to provide hope and reassurance in the face of challenges. Some of the participants also described strangers providing support. Speaking emotionally, a grandmother of a four-year-old girl said:

Neighbours are always there for me [wiping tears with her cloth, then quiet for a moment]. They help me a lot [crying]. There’s a good neighbour in the house you’ve just passed over there, they give me money for sugar, some buy me sugar, that’s how it is.

Negative societal norms and attitudes create barriers to accessing essential services. This disabling environment is socially created and manifested through prejudice from nuclear-family members, the extended family, the public, and institutions. A mother of a nine-year-old girl recounted an example of this:

When some other doctors came, they insulted me, they said, ‘these mothers’ biggest problem is that they squeeze their children when giving birth, that’s why they give birth to children like these [disabled children]’ […] I told them to just let me go, the nurses came back and begged me not to go. But I had to leave for the safety of my child.

Similarly, the mother of a nine-year-old boy who lives in a neighbourhood nearby recounted the following:

At the beginning, people used to say that we [the family] had sacrificed the child for taboo things or rituals to become wealthy, it reached a point that it really made us so sad. We were so affected by that and kept the child inside the house. We were afraid to take the child outside.

### Self-esteem far beyond reach

Self-esteem is a higher-level human need. In this study, families expressed their sense of self-esteem in mixed terms. Family members, particularly the primary carers of disabled children, exhibited only partial self-appreciation when acknowledging their accomplishments. This observation suggests that the fulfilment of self-esteem was beyond the reach of these families. Conversely, the study findings also reveal instances of disrespect from e.g. healthcare providers, as attested to by the mother of a four-year-old girl:

One of the things that scared me was taking my baby to the hospital when she was burnt, and the doctors’ humiliations, yes. [speaking very sadly]

Another aspect of human self-esteem is the aspiration for freedom. This can involve the ability to visit friends, travel, and work for an income. Some of the participants described as a sense of captivity and imprisonment in caring for their disabled children.

The mother of a six-year-old boy said:

Sometimes you do not have someone to leave the child with, so you often fail to participate in social gatherings, even funerals. I often do not participate at all because of the child.

### Dreaming of self-actualisation

Human beings desire to become what they wish to be, and ‘self-actualisation’ is the uppermost need in Maslow’s hierarchy. The participants described difficulties in parenting their other children, and sometimes compromising the care of other family members in order to attend to the needs of the child or adolescent with a disability. Finding educational opportunities for children and adolescents with physical disabilities is an ongoing challenge. However, these unfulfilled desires and expectations create uncertainty about the future. One mother of an 18-year-old female said:

I don’t know how to put this […] If she [the disabled teenager] had been successful with school, you would find that she would have taken a big step. But instead, she is here at home.

Concerns were also expressed regarding the future wellbeing of children in the event of the parents’ own absence or passing. Primary carers often find it challenging to pursue their own dreams and aspirations, and achieving desired goals such as successful parenting and providing education for their children remain an elusive dream. Moreover, financial stability (in the sense of e.g. savings) is often overshadowed by more immediate needs, such as securing food and healthcare. Consequently, families in these circumstances often experience feelings of helplessness, desperation, and uncertainty.

### Adaptive adjustment

‘Adaptive adjustment’ is a term that describes the actions taken by the interviewees to tackle challenging circumstances, such as avoidance, accepting reality and seeking alternative solutions. Some of the participants described acknowledging and accepting the reality of their situation when trying to meet basic needs. For example, when seeking healthcare, they may opt for private or larger hospitals in order to receive better care; when leaving the house, they may lock their children inside for added security. The participants also described avoiding disrespectful or discriminatory environments by distancing themselves from these. The stories of the participants illustrate a continuous search for alternative solutions to social, cultural, and economic hardships. For unfulfilled self-actualisation needs, they may choose alternative ways of achieving their desires, such as providing informal education at home instead of sending their disabled children and adolescents to schools.

## Discussion

This study provides a theoretical analysis of the needs of and challenges faced by families in relation to caring for children and adolescents with physical disabilities in north-eastern Tanzania. The needs expressed included resources, care, services, and life aspirations. Insufficient resources for meeting these needs are a significant challenge in Tanzania [42]. The challenges described were associated with insufficient financial resources for basic needs (food, clothes, transportation) and healthcare. An unsupportive environment imposes hardships on family members, especially primary carers. Insufficient support has been linked to the poor mental well-being of family members [11], who worry about fulfilling basic needs rather than their life aspirations. A significant struggle is that of families to meet healthcare needs, and healthcare expenditure and the absence of health insurance increase the burden on families. Consistent findings of poor living condition for people with disabilities are reported in Malawi, Zambia, and Zimbabwe [43–45]. These challenges affect the social inclusion of children and adolescents with physical disabilities.

Families also face challenges due to multiple interrelated societal barriers. The findings of this study show that families must deal with various negative perspectives on the part of both institutions and society. Uncharitable beliefs and stigma towards disability are commonly reported in resource-poor settings. In Tanzania, similar findings are reported by the mothers of children with disabilities [11]. Negative beliefs could be attributable to poor awareness of disability. The participants spoke about barriers to transportation, access to which is critical for daily life. A scoping review in low-income settings has reported similar findings regarding transportation [46]. The lack of government funding for infrastructure contributes to these challenges.

The findings also exemplify the social model of disability [47], which links disability to societal barriers; this is in contrast to the medical model, which focuses on individual deficits. However, there are arguments that the social model of disability sometimes disregards individual experiences [47]. Both models present challenges for children and adolescents with disabilities, as well as their families. The World Health Organization proposes a comprehensive biopsychosocial perspective in order to account for the individual and societal factors that contribute to disability [3].

One of the key aspects of Maslow’s hierarchy of needs is that one must meet the lower-level needs before moving to the higher ones [40]. However, this approach tends to fragment human needs. The findings of this study indicate that families experience multiple needs simultaneously, and these are often contradictory. Previous research has reinforced the view that human needs are interdependent, and occur concurrently [48]. In families caring for a physically disabled child or adolescent who requires extra resources, needs emerge and must be adapted to. The findings also suggest that families adapt to challenging needs as part of a dynamic, interactive process. Agrawal [48] argues that Maslow’s hierarchy of needs theory is difficult to generalise from and may even have elements of inequity due to the conception of ‘lower’ and ‘higher’ levels.

The families interviewed during this study appeared to face reality and accept their life situations. They also tended to avoid difficult circumstances. Families often search for alternative solutions to difficulties, such as changing their lifestyles. Abery [49] proposed a model of short-term actions and adjustments and long-term strategies for adapting to and accommodating challenges. The outcomes of these adaptations can differ based on the family’s capacity, available resources, and social influences. This study demonstrates that a supportive environment of social and cultural structures promotes empowerment and facilitates fulfilment of family needs. Support from the nuclear family is fundamental. An extended family network that complements the nuclear family is important. The benefits of extended family support are reported in resource-poor settings [50]. Carers for children with disabilities can be provided with relief and e.g. go to work through the presence of support from extended family. These unstructured sociocultural support mechanisms are not available consistently. A qualitative study of carers for children with disabilities in South Asia reported limited evidence regarding the scope and reliability of extended-family support [51]. The present study underscores the importance of the support of the nuclear family in the Tanzanian context. Such networks have demonstrably positive outcomes on the health of individuals, as postulated in social-capital frameworks [36]. Despite the existence of contradictory views, cultural values and social structures are core ingredients of enhanced resilience.

The study also covered unsupportive relationships and encounters with nuclear and extended family. In most cases, misconceptions led to oppression of and blame against the mother. A qualitative study conducted in Moshi, Tanzania, reported that family members, including fathers, leave when they discover that a child has a disability [11,52]. A UNICEF report about care reform in Africa showed that extended families stigmatise children with disabilities and offer no support [53]. The similarity of those findings to the ones presented in this study suggests poor societal awareness regarding disability. The unsupportive dimension creates disabling experiences, hindering service-seeking behaviours and inclusion.

This study revealed that the interviewed members of families with physically disabled children often fail to fulfil their self-esteem and self-actualisation needs. These challenges predispose family members to poor physical, social, and psychological wellbeing, which can manifest as worry, hopelessness, unhappiness, and uncertainty. It is therefore crucial to promote favourable coping mechanisms [54]. Tanzania has comprehensive policies relating to people with disabilities: the 2010 Disability Act was a promising step towards helping people with disabilities and their families [23], as Sections II and III of the Act committed the government to promoting the wellbeing of people with disabilities by guaranteeing equality and protection against discrimination. Furthermore, the Act stipulated that the government, in collaboration with other stakeholders, should promote awareness of rights and the potential of people with disabilities. Section VII of the Act elaborated on the provision of healthcare and rehabilitation services. Improving the implementation of the Act would better support families in caring for children and adolescents with physical disabilities. In addition, interventions such as community-based rehabilitation could be expanded in Tanzania through governmental funding to coordinate and implement programmes. The provision of services is described in Section VII, Subsection 26 the 2010 Disability Act [23], where it is stated that the government will ensure the availability of rehabilitation services and basic equipment for people with disabilities in their respective localities [22,23].

In this study, all of the families contacted were resource-poor. Additional demands on food increase the need for financial and time resources. Mothers, who are often carers, are particularly affected. Unfortunately, this vicious cycle is self-perpetuating, and there is a reluctance to have more children due to the hardships of caregiving and social barriers. Essential healthcare services are often expensive and inaccessible. UNICEF reports similar findings, whereby the families of children with disabilities experience additional costs and increased poverty [16,55]. The findings of this study provide context and perspective for addressing the needs of families who care for children and adolescents with physical disabilities. These needs are comparable to, but more complex than, Maslow’s hierarchical framework.

This study underscores the importance of improving awareness of disability inclusion among the public in Tanzania. Raising awareness will dissuade unsupportive societal actions and promote the sustainability of sociocultural protection, i.e. family networks. Additionally, the government and other stakeholders should promote the inclusion of children with disabilities by establishing supportive systems. Inclusive systems are outlined in the 2004 and 2010 Disability Acts, covering various aspects, including human rights, settlement, health, education, and transport [22,23]. In addition, involving religious and local-government leaders could enhance the promotion of inclusive attitudes towards disabilities within families and society. The concept of inclusivity should be integrated into training programmes for healthcare workers in Tanzania to enhance the capacity of the workforce to provide equitable healthcare services for individuals with disabilities.

In terms of healthcare expenditure, an improved health-insurance scheme funded by the government could relieve some of the financial burden on families. Meeting the educational needs of children and adolescents with disabilities is a crucial priority for families, and the lower rates of school enrolment for children with disabilities than children without disabilities in Tanzania show that further government efforts are required. Such efforts should include schemes aimed at training teachers so that they could teach children with special educational needs. Government support should be strengthened to provide resources for disabled students to support their learning. In the Tanzanian context, additional financial assistance, i.e. coverage of school fees for disabled children, should be considered.

This study has several strengths. Lincoln and Guba’s criteria for credibility, transferability, dependability, and confirmability were used to ensure trustworthiness [56]. To strengthen credibility, purposive sampling was used to select participants with diverse characteristics (see Table 1). The first author’s professional background in rehabilitation/physiotherapy and prior experience in community-outreach programmes in Tanzania enhanced the theoretical understanding of the contextual issues. Three of the authors are Tanzanian. All of the participants verified the interview summaries and approved their use in this research. The multidisciplinary research team held regular debriefings and reflections. Comparative, iterative methods were used to analyse and interpret the data, using field-observation notes, memos, and transcript text. Memos were used during data collection and analysis to ensure confirmability. A detailed account of the data-handling and -analysis process is outlined in Table 2.

The study had some limitations, however. The findings rely heavily on the views of the interviewed mothers, but any family has multiple members. The extent to which primary carers (mostly mothers) represented the ‘family perspective’ was not captured in the data. Future qualitative research is needed to provide in-depth analyses of the views of fathers, other carers, and disabled adolescents themselves in order to enrich the understanding of the complexity of families.

## Conclusion

In northeastern Tanzania, families who care for children and adolescents with physical disabilities develop adaptation strategies, including avoidance and reality acceptance. They can also seek alternative ways of coping. A sustainable support system of social networks is crucial to meeting basic needs and ensuring safety. Improving the level of awareness of the importance of social inclusion can foster social support for disabled people and reduce negative beliefs around their conditions. The government, in collaboration with other stakeholders, should promote awareness of the rights, abilities, contributions, and potential of people with disabilities. Awareness campaigns should focus on improving knowledge about disability inclusion. Improving the implementation of the 2010 Disability Act would involve a political commitment to mitigating the challenges associated with caring for children and adolescents with disabilities. The government should review the health-insurance tariffs, and consider a comprehensive that is package tailored to people with disabilities and affordable for poor families.
